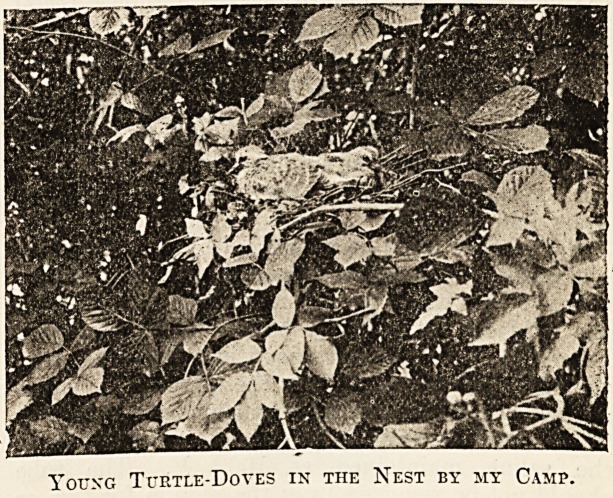# A Bird-Watcher's Camp—II

**Published:** 1910-09-24

**Authors:** 


### The Practitioner's Relaxations.

### A BIRD-WATCHER'S CAMP—II.

I have referred to three cuckoos I heard at dawn on my first morning in camp. I was able to count them without seeing them, partly by the different directions from which the calls came, and partly by a difference in the pitch of the voices of two of the birds.

Recently I again observed this difference in the calls of two cuckoos answering each other, and, although it is generally held that only the male bird utters the call from which the name is derived, I became convinced that the more highly-pitched "cuckoo" was uttered by the female. I watched the two birds together for about fifteen minutes as they chased each other from tree to tree, in love rather than in anger, and during this time I frequently heard the well-known rippling note of the female.

None of the three cuckoos I heard on my first morning in camp showed any signs of a stammer. "In June he changes his tune" says the old rhyme, referring to the stammer which generally affects cuckoos soon after the commencement of June and is the prelude to their return to silence. But I heard cuckoos as late as June 22, and one as late as the 26th calling in full, clear voice. On the former date I heard several at 3 a.m.; the bird I heard on the 26th was calling at 8.30 p.m. All wild birds sing their best in the early morning and late evening or during and after rain, while in the noonday heat of a fine summer day they are generally silent. It may be that the oppressive heat of the midday sun affects their vocal cords, and that the cuckoos which called so clearly and loudly in the cool mornings and evenings would have broken down in the attempt to repeat the performance at other times of the day.

There are few subjects upon which I find people more prone to dogmatise than the question whether the lower animals are capable of thinking, or, to put the matter more precisely, of any sort of reasoning process, however limited. It is, perhaps, because the question is so difficult that it is treated so dogmatically. I hope to avoid this error. One is almost compelled to attribute, for instance, the architectural skill of birds to instinct. But instinct does not seem to me to be incompatible with intelligence, and having spent a good deal of my leisure time in the society of birds, if I may so express it, I am inclined to credit them with a moderate degree of reasoning power, and when they behave most foolishly I fancy their nerves rather than their intelligence should be blamed.

I gave a good deal of attention to one of the many pairs of willow-wrens near my camp, and they met me with what appeared to me to be reasoned mistrust. They had a nest with young in some rough grass, and I endeavoured without success to photograph them while they fed their young. I got them used to a dummy camera without difficulty, and when this was replaced by the real camera they were not above using it as a perching-place. I had a 20-feet indiarubber tube attached to the shutter of the camera, and I rigged up a hiding-place for myself just near enough to enable the bulb of the tube to reach it. They appeared to regard the hiding-place and the tube with unconcern, and fed their young as usual. They did not appear to take any special notice of me as I moved about in the neighbourhood of the shelter. But when I was in the hiding-place and connected up, so to speak, with the camera by the nest nothing would induce them to approach it. They visited my hiding-place frequently, hopping all over it, calling anxiously and peering in through every crack to see what I was doing, but they would not face the camera. I could not prevent them from seeing me go into my shelter because it was in the middle of an open field. On several successive days I lay there till it was too dark to hope to get a photograph, or till I was afraid of killing the young by starvation, always with the same result, and at last I gave up the attempt in despair. I could not avoid the conclusion that these birds had, however erroneously, thought the matter out, and had decided that a camera by itself was a harmless, inanimate object, but that a camera connected up with a man was probably a dangerous machine. And it must be remembered that they came boldly, time after time, to inspect me. Evidently, therefore, they were not acting under the impulse of mere bodily terror, but were soberly and warily investigating a suspicious object.

Most people have had some experience of the devices birds will employ to lure an intruder away from their young. The most common device, and one very hard to account for by mere instinct, is to feign an injury and flutter away in front of the intruder till he has been drawn away from the place where the young are concealed. A nightingale which had a brood near my camp adopted a different method. The young birds were full-fledged when I found the nest, and I decided to take a photograph of them without delay. Before going to fetch my camera, which was not far off, I cautiously displaced some of the undergrowth in front of the nest in order to expose it fully to the camera. Having made my preparations without alarming the young birds, as far as I could see, I started to fetch the camera, but I turned, after I had taken a few steps, to make sure that I had marked the spot satisfactorily. One of the parent birds was by the nest and appeared to be pecking the young viciously. He left them when I approached, and hastily deciding that I must have been mistaken, I hurried off to fetch the camera. When I returned a few minutes later the nest was empty, the young had completely disappeared, and the only indication of the presence of nightingales was a harsh warning note in the bushes hard by. Clearly my first impression was correct, and the old bird had driven the young from the nest where they seemed to him to be no longer safe. He was cruel to be kind, and I think he acted intelligently.

I must refer to one more manifestation—as I regard it—of intelligence in birds, though I admit that the exponents of the theory of inherited instinct will hardly be convinced by it. In the bushes close to my tent there was a turtle-dove's nest. The turtle-dove's nest is a shallow, fragile structure of twigs and roots placed, as a rule, not more than 10 or 15 feet from the ground. The nest in question was about 10 feet up in an elder bush overgrown with brambles, which screened it from view. In the accompanying photograph of the nest, taken at a later date than that with which I am at present concerned, the brambles have been set aside. I visited the nest frequently while the bird was sitting, and she generally left it when I was some three or four yards away. One evening, however, the wind blew so severely that I doubted whether my tent would stand up, and it occurred to me that the turtle-dove's nest was somewhat precariously placed for such weather. I approached the nest very cautiously in full view of the bird; the bush was swaying violently, and the nest was frequently at

Young Turtle-Doves in the Nest by my Camp.

such an angle that the eggs, left to themselves, must inevitably have rolled out of it. The bird was not merely sitting, she was clinging on with an effort, and when I stood almost immediately under the nest she refused, although she watched me with an anxious eye, to risk disaster to her eggs by leaving the nest. I dared not put her to any severer test, so I retired. During the night the wind fell, and in the morning I paid another visit to the nest. The bird left the nest as usual when I was some yards away.

## Figures and Tables

**Figure f1:**